# Supplementary material for: A Critical View of the Application of the APEX Software (Aqueous Photochemistry of Environmentally-Occurring Xenobiotics) to Predict Photoreaction Kinetics in Surface Freshwaters
Source: Molecules. 2019 Dec 18;25(1):9. doi: 10.3390/molecules25010009 (PMC7017383; doi:10.3390/molecules25010009)
Supplement: Supplementary file 1 [file molecules-25-00009-s001.zip › 3.2.4_gcc-4.4.0.zip!/3.2.4_gcc-4.4.0/3.2.4_gcc-4.4.0/doc/octave/html/interpreter/Structures-with-Mex_002dFiles.html]

Structures with Mex-Files - Untitled


Next: Sparse Matrices with Mex-Files,
Previous: Cell Arrays with Mex-Files,
Up: Mex-Files


---

#### A.2.5 Structures with Mex-Files

The basic function to create a structure in a mex-file is
`mxCreateStructMatrix`, which creates a structure array with a two
dimensional matrix, or `mxCreateStructArray`.

```
     mxArray *mxCreateStructArray (int ndims, int *dims,
                                   int num_keys,
                                   const char **keys);
     mxArray *mxCreateStructMatrix (int rows, int cols,
                                    int num_keys,
                                    const char **keys);
```

Accessing the fields of the structure can then be performed with the
`mxGetField` and `mxSetField` or alternatively with the
`mxGetFieldByNumber` and `mxSetFieldByNumber` functions.

```
     mxArray *mxGetField (const mxArray *ptr, mwIndex index,
                          const char *key);
     mxArray *mxGetFieldByNumber (const mxArray *ptr,
                                  mwIndex index, int key_num);
     void mxSetField (mxArray *ptr, mwIndex index,
                      const char *key, mxArray *val);
     void mxSetFieldByNumber (mxArray *ptr, mwIndex index,
                              int key_num, mxArray *val);
```

A difference between the oct-file interface to structures and the
mex-file version is that the functions to operate on structures in
mex-files directly include an `index` over the elements of the
arrays of elements per `field`. Whereas the oct-file structure
includes a Cell Array per field of the structure.

An example that demonstrates the use of structures in mex-file can be
found in the file mystruct.c, as seen below

```
```
     #include "mex.h"
     
     void
     mexFunction (int nlhs, mxArray* plhs[], int nrhs, 
     	     const mxArray* prhs[])
     {
       int i;
       mwIndex j;
       mxArray *v;
       const char *keys[] = { "this", "that" };
     
       if (nrhs != 1 || ! mxIsStruct (prhs[0]))
         mexErrMsgTxt ("expects struct");
     
       for (i = 0; i < mxGetNumberOfFields (prhs[0]); i++)
         for (j = 0; j < mxGetNumberOfElements (prhs[0]); j++)
           {
             mexPrintf ("field %s(%d) = ", 
                        mxGetFieldNameByNumber (prhs[0], i), j);
             v = mxGetFieldByNumber (prhs[0], j, i);
             mexCallMATLAB (0, 0, 1, &v, "disp");
           }
     
       v = mxCreateStructMatrix (2, 2, 2, keys);
     
       mxSetFieldByNumber (v, 0, 0, mxCreateString ("this1"));
       mxSetFieldByNumber (v, 0, 1, mxCreateString ("that1"));
       mxSetFieldByNumber (v, 1, 0, mxCreateString ("this2"));
       mxSetFieldByNumber (v, 1, 1, mxCreateString ("that2"));
       mxSetFieldByNumber (v, 2, 0, mxCreateString ("this3"));
       mxSetFieldByNumber (v, 2, 1, mxCreateString ("that3"));
       mxSetFieldByNumber (v, 3, 0, mxCreateString ("this4"));
       mxSetFieldByNumber (v, 3, 1, mxCreateString ("that4"));
     
       if (nlhs)
         plhs[0] = v;
     }
```
```

An example of the behavior of this function within Octave is then

```
     a(1).f1 = "f11"; a(1).f2 = "f12";
     a(2).f1 = "f21"; a(2).f2 = "f22";
     b = mystruct(a)
     => field f1(0) = f11
         field f1(1) = f21
         field f2(0) = f12
         field f2(1) = f22
         b =
         {
           this =
     
           (,
             [1] = this1
             [2] = this2
             [3] = this3
             [4] = this4
           ,)
     
           that =
     
           (,
             [1] = that1
             [2] = that2
             [3] = that3
             [4] = that4
           ,)
     
         }
```
